# Differential responses to double-stranded RNA injection and feeding in Mormon cricket (Orthoptera: Tettigoniidae)

**DOI:** 10.1093/jisesa/iead063

**Published:** 2023-08-01

**Authors:** Seema Rana, Changsun Kang, Julianne Allred, Jorge Humberto Medina-Duran, Alyssa Canova, Danielle Sherry, Derek A Woller, Dongin Kim, Hojun Song

**Affiliations:** Department of Entomology, Texas A&M University, College Station, TX, USA; Department of Pharmaceutical Sciences, The University of Oklahoma Health Sciences, Center, Oklahoma City, OK, USA; Department of Entomology, Texas A&M University, College Station, TX, USA; Department of Entomology, Texas A&M University, College Station, TX, USA; Department of Entomology, Texas A&M University, College Station, TX, USA; Department of Entomology, Texas A&M University, College Station, TX, USA; USDA-APHIS-PPQ-Science & Technology-Insect Management and Molecular Diagnostics Laboratory (Phoenix Station), Phoenix, AZ, USA; Department of Pharmaceutical Sciences, The University of Oklahoma Health Sciences, Center, Oklahoma City, OK, USA; Department of Entomology, Texas A&M University, College Station, TX, USA

**Keywords:** RNAi efficiency, knockdown, oral delivery, dsRNA-encapsulating nanoparticles

## Abstract

The Mormon cricket, *Anabrus simplex*, is a flightless katydid, one of the major devastating rangeland pests in several states of the western United States. During the past few years, their sudden and periodic outbreaks into massive migratory bands caused significant economic losses to the rangeland forage and agricultural crops, particularly grain crops. Current population management methods rely heavily on broad-spectrum chemical insecticides, which could be toxic to nontargets, and even the targeted species might develop resistance in the long run. Therefore, we assessed the potential of RNA interference (RNAi)-based alternative management strategies that could supplement the current methods. In insects, RNAi efficiency varies with the method of double-stranded RNA (dsRNA) delivery. We tested 2 different methods of dsRNA delivery: injection and oral feeding of dsRNA. The results showed that Mormon crickets are sensitive to injection of dsRNA in a dose-dependent manner, but refractory to the oral feeding of dsRNA. Further, we confirmed the high nuclease activity in the insect midgut. In order to protect the dsRNA from the dsRNase activity and facilitate its uptake in the midgut, we encapsulated dsRNA inside poly lactic-co-glycolic acid (PLGA) nanoparticles and studied its release kinetics and RNAi efficiency by oral feeding. The release kinetics clearly suggested that the PLGA nanoparticle permeates from the insect digestive system to the hemolymph; however, it failed to induce an efficient RNAi response of the targeted genes. In conclusion, our findings suggest the different responses to dsRNA delivery methods in Mormon crickets, and further investigations involving dsRNA stability and its uptake mechanism are required to use RNAi as an alternative Mormon cricket population management strategy.

## Introduction

The Mormon cricket, *Anabrus simplex* Haldeman, 1852 (Orthoptera: Tettigoniidae), is a flightless shield-backed katydid, which is considered a major rangeland pest in the western United States (Cowan 1929, Wakeland 1959, Cowan and Wakeland 1962, USDA APHIS 2019). Under favorable conditions, Mormon crickets can increase in population size to reach the outbreak level, competing with available human and livestock food resources (USDA APHIS 2019). During outbreaks, these insects can form large migratory bands, which can contain billions of individuals, that migrate long distances, up to 2 km per day, by walking and jumping (Cowan 1929, Wakeland 1959, Simpson et al. 2006). These migratory bands can consume every vegetation or crop present along the way, causing significant ecological and economic losses to the rangeland vegetation or the cropland, especially the grain crops (USDA APHIS 2019). Currently, federal programs, such as the US Department of Agriculture (USDA) Animal and Plant Health Inspection Service (APHIS) Rangeland Grasshopper and Mormon Cricket Suppression Program, mostly employ traditional chemical insecticides for population management, but are greatly interested in viable alternatives. The most common chemical insecticides used for management are carbaryl and diflubenzuron, which are broad-spectrum insecticides affecting the insect nervous system (Tomlin 1994) and growth (US EPA 1997), respectively. However, chemical insecticides have their own associated risks, such as resistance evolution in the target insects and other toxic effects on nontarget species, such as other arthropods. This necessitates finding an alternative strategy to supplement, or possibly even replace, the present Mormon cricket management methods.

RNA interference (RNAi) is a conserved and naturally occurring defense mechanism in eukaryotes, in which double-stranded RNAs (dsRNAs) down-regulate the expression of a target gene in a sequence-specific manner (Fire et al. 1998). The ability of RNAi to knock down expression levels of any target gene has quickly led scientists to apply this technique for insect pest management, prompting hundreds of studies that describe various aspects of developing RNAi-based insecticides (Gordon and Waterhouse 2007, Price and Gatehouse 2008, Huvenne and Smagghe 2010, Xue et al. 2012, Gu and Knipple 2013, Scott et al. 2013, Zotti and Smagghe 2015, Joga et al. 2016, Cooper et al. 2019, Vogel et al. 2019, Zhu and Palli 2020). However, insects belonging to different insect orders have shown variable RNAi responses (Cooper et al. 2019). For Orthoptera, to which the Mormon cricket belongs, RNAi responses via injection appear systemic. A number of studies on the desert locust (*Schistocerca gregaria*), the migratory locust (*Locusta migratoria*), as well as various cricket species, have shown that RNAi via injection works very well in these insects (Wynant et al. 2012, Luo et al. 2013, Song et al. 2017, Spit et al. 2017). As the first attempt to develop RNAi-based control methods for the Mormon cricket, we previously generated a de novo transcriptome for this species, developed dsRNAs of housekeeping genes, quantified RNAi efficiencies by injection, and demonstrated variable gene-specific and tissue-specific RNAi responses (Hoang et al. 2022).

Typical laboratory experiments for developing RNAi utilize injection of dsRNA directly into insects, but this is perhaps the least viable option for delivering dsRNA to pest insects on a large scale. Some studies have demonstrated that direct spraying dsRNA onto the insects (known as topical application) can penetrate insect integument and induce RNAi response in mosquitoes (Pridgeon et al. 2008), caterpillars (Wang et al. 2011), and hemipteran insects (El-Shesheny et al. 2013, Killiny et al. 2014, Niu et al. 2019, Yan et al. 2020), but this, too, is not a viable delivery option for large-scale pest management. Transformative RNAi, which utilizes transgenic plants expressing dsRNA (Zha et al. 2011, Zhang et al. 2015), can be a powerful approach to selectively suppress insect pests. Only recently have RNAi-based genetically modified hybrid corns (SmartStax Pro and VT4PRO, Bayer AG) been approved by the Environmental Protection Agency (EPA) to manage coleopteran species (below-ground corn rootworms, *Diabrotica virgifera* and *Diabrotica barberi*) and other above-ground corn insect pests (US EPA 2017). However, transgenic approach narrowly targets specialist insect pests and requires creation of genetically modified crops that must go through many regulatory hurdles from governmental agencies and the scrutiny of the general public in terms of reception (Joga et al. 2016). The most viable option for delivering dsRNA to control insect pests in the field is the formulation that allows oral feeding. Many studies have reported the application of dsRNA onto the plant leaves, which allowed insects to feed on the treated leaves, thereby delivering dsRNA into the digestive system of the target pests (Whyard et al. 2009, Joga et al. 2016, San Miguel and Scott 2016, Zhu and Palli 2020). dsRNA can also be incorporated into baits or artificial diets (Wynant et al. 2012, Zhu and Palli 2020).

For orthopteran insects, oral feeding of dsRNA has consistently failed to elicit RNAi responses (Wynant et al. 2012, 2014, Luo et al. 2013, Ren et al. 2014, Spit et al. 2017) due to the potent activities of double-stranded ribonucleases (dsRNases), specifically *dsRNase2* in the midgut, where the orally fed dsRNAs pass through and get degraded. In fact, dsRNases are known to be abundantly present in the midgut of many insects (Zhu and Palli 2020). Although it is possible to knock down the expression of dsRNases through RNAi through injection (Wynant et al. 2014), there is currently no practical solution to overcome this biological process if oral feeding is to be the main mode of delivery.

Recently, several insect studies have used various nanoparticles to encapsulate dsRNA in an attempt to prevent it from being degraded by dsRNases upon oral delivery to insects. What follows are examples of such studies, starting with chitosan-based nanoparticles that encapsulated dsRNA demonstrated a 66% reduction in transcript levels in mosquito larvae when combined with diflubenzuron application (Zhang et al. 2010, 2015b). In Asian corn borers, (*Ostrinia furnacalis*), cationic core-shell fluorescent nanoparticle that encapsulated dsRNA, fed through insect diet, resulted in growth and development defects, eventually leading to death (He et al. 2013). Guanylate polymers complexed with dsRNA were shown to protect the dsRNA from degradation for up to 30 h and enhanced its cellular uptake in beet armyworm (*Spodoptera exigua*) (Christiaens et al. 2018). Dendrimer-coated carbon nanotubes (CNTs) resulted in stable dsRNA and increased mortality in red flour beetle (*Tribolium castaneum*) (Edwards et al. 2020). Encapsulation of dsRNAs with block copolymer poly (ethylene glycol)-polylysine (thiol) led to improved oral RNAi efficiency in the migratory locust (Lu et al. 2022). What these studies have collectively demonstrated is the potential for nanoparticle encapsulation of dsRNA to overcome the issues of RNAi efficiency.

In this study, we first compared RNAi efficiencies in the Mormon cricket using 2 dsRNA delivery methods: injection and oral feeding. For these comparisons, we specifically targeted housekeeping genes (Annexin IX (*AnnIX*), Ribosomal protein L5 (*RpL5*), Glyceraldehyde-3P-dehydrogenase (*GAPDH*)), double-stranded ribonuclease 2 (*dsRNase2*), and chitin synthase 1 (*CHS1*)). In order to prevent dsRNA degradation, we then explored the utility of polylactic-co-glycolic acid (PLGA), a polymer approved by the Food and Drug Administration (FDA) for drug and protein delivery, which can be synthesized by co-polymerization of glycolic acid (GA) and lactic acid (LA) (Wise et al. 1976, Gilding and Reed 1979, Makadia and Siegel 2011), to encapsulate dsRNAs (Makadia and Siegel 2011). We characterized the release kinetics of the PLGA nanoparticles and established their permeability across the digestive tract. Furthermore, we tested the hypothesis of whether dsRNA-encapsulating nanoparticles could elicit RNAi responses in Mormon crickets. Finally, we performed a dose-dependent response feeding assay with dsRNA-encapsulating nanoparticles to test their effects on the RNAi efficiencies.

## Materials and Methods

### Insect Collection and Rearing

The Mormon crickets were originally collected from an outbreak population near Murphy, Idaho (43.036031, −116.721747 WGS84) on 4 June 2021, and temporary colonies were maintained in the USDA-APHIS-Plant Protection and Quarantine (PPQ)-Field Operations facility in Boise, Idaho. For part of the experiments, temporary colonies were also maintained in the Department of Entomology at Texas A&M University, College Station, Texas. Specimens were kept in cages (55 × 35 × 30 cm) in a walk-in incubator, set at 20°C, and fed daily with Cheerios, Romaine lettuce, and fish flakes (hereafter, collectively mentioned as MC food), as described in (Hoang et al. 2022).

### dsRNA Synthesis

To synthesize dsRNAs, we first extracted total RNA from a single adult head or thorax tissue using a Trizol method (Chomczynski and Mackey 1995), followed by purification with a DNase treatment using an RNeasy mini kit (Qiagen, Hilden, Germany). The isolated RNA was reverse-transcribed using an iScript cDNA synthesis kit (Bio-Rad, Hercules, CA). Gene-specific primers (Table 1) were designed from the orthologs identified in the assembled transcriptome data (Hoang et al. 2022), and additional T7 promoter sequences (TAATACGACTCACTATAGGGAGA) were added to the 5’end of the primers. Polymerase chain reaction (PCR) was carried out with the following conditions: 94°C for 2 min; 10 cycles of 94°C for 30 s, 55–60°C for 30 s, 72°C for 1:30 min followed by 25 cycles of 94°C for 30 s, 69°C for 30 s, 72°C for 1:30 min; and 72°C for 2 min. The amplified PCR products were purified with a Monarch PCR and DNA clean-up kit (New England Biolabs, Ipswich, MA) following the standard protocol in the manual. The purified PCR products were used for dsRNA synthesis with a MEGAscript RNAi kit (Thermo Fisher Scientific, Waltham, MA) following the standard manual protocol. Green Fluorescent Protein (*GFP*) dsRNA was amplified from TOPO GFP plasmid (Addgene, Watertown, MA) and used as a non-target control in the experiments.

**Table 1.** Primers used in this study

Gene name (accession number)	Sequence(5ʹ–3ʹ)	Amplicon size (bp)
ds*GAPDH* (ON402768)	F:taatacgactcactatagggCTTCAAGGGAGAGGTGAAAGC R:taatacgactcactatagggGAGCCAGACAGTTGGTAGTGC	311
ds*CHS1* (OQ584308)	F:taatacgactcactatagggTAGCACCATCGACGAAGCAG R:taatacgactcactatagggCACCATTGGACCTGAGCCTA	400
ds*dsRNase2* (OQ683315)	F:taatacgactcactatagggagaAACTACCTGTCGCCGTTCAG R:taatacgactcactatagggagaCTTCTTCCCCAGATCGGCAG	421
ds*AnnIX* (ON402772)	F:taatacgactcactatagggagaTTTTGGCTGTGATGAGCAAG R:taatacgactcactatagggagaCGTTTGAAGTGACCAGATGC	364
ds*RpL5* (ON402766)	F:taatacgactcactatagggagaCCTCCGTCTGATCTCTCAGG R:taatacgactcactatagggagaTCCCCGACCACTTTCTACAG	321
qPCR *GAPDH*	F:CAACTGTCTGGCTCCTCTGG R:CCATCACGCCACAACTTTCC	135
qPCR*CHS1*	F:TGTGTGCTGTGTAGTCCAGG R:GAGCCTCATCCGATCTGGTG	100
qPCR *dsRNase2*	F:ACTGTGTCCTGATGTGAGCG R:AGATGTTGGGTATGCTGGGC	128
qPCR *AnnIX*	F:CAATTTTGGTGACCCGTAGC R:CAATAGCCAGCAGTCCCTTC	135
qPCR*RpL5*	F:GGTGCCAGAGTGTTTGGTG R:ACTCTTTTGATTCCGCATCG	103
qPCR*EF1α* (ON402770)	F:CAAGATGGGCTGGTTTAAGG R:CTCAGTAGGCCTGGAAGGTG	112

### RT-qPCR Analysis

For all quantifications of the RNAi experiments, we extracted total RNA from 3 to 5 individual head and thorax tissues separately, and each individual was considered a biological replicate. The RNA concentration and purity were assessed using a spectrophotometer (DeNovix DS-11) and reverse-transcribed to produce cDNA using an iScript cDNA synthesis kit (Bio-Rad, Hercules, CA). The diluted cDNA (1:10 dilution) was used as a template for qPCR amplification using gene-specific primers (Table 1) on CFX Connect Real-Time PCR Systems (Bio-Rad, Hercules, CA). The 20 µl qPCR reaction mixture consisted of 10 µl of SYBR Green supermix (Bio-Rad, Hercules, CA), 500 nM primers, and nuclease-free water to make up the final volume. The thermal cycling included initial denaturation for 3 min at 95°C followed by 40 cycles at 95°C for 10 s and 60°C for 30 s. Each reaction was performed with 3–5 biological replicates and 2 technical replicates. A no-template control (NTC) and a no-reverse transcriptase control (NRT) were included to check for reaction mixture contamination and genomic DNA contamination, respectively. The relative gene expression was analyzed using the CFX Manager 2.1 (Bio-Rad, Hercules, CA) following the 2^−∆∆*C*T^ method. The results were normalized against the reference genes, Elongation factor 1α (*EF1*α), Ribosomal protein L5 (*RpL5*), or Actin 5C (*Act5C*). Statistical differences were derived from the Student’s *t*-test or Wilcoxon test based on normality and variance assumptions at significance levels of 0.05 (in R, Version 4.2.1).

### dsRNA Dose-dependent Response Assay

To study dose-dependent responses, dsRNA of the target gene, *GAPDH*, or the control (*GFP*) were diluted to various dsRNA concentrations (200, 500, and 1,500 ng/µl) using the locust saline solution (500 ml: 4.383 g NaCl; 0.094 g CaCl_2_; 0.373 g KCl; 0.203 g MgCl_2_; 0.168 g NaHCO_3_; 15.403 g sucrose; 0.946 g trehalose; pH 7.2). The injection was carried out using a Hamilton precision microinjection pump (700 series, 705RN, 50 µl, Sigma–Aldrich) with a 22-gauge needle. The variable doses (2, 5, and 15 µg) of dsRNAs were injected into 10 adult females (per dose per gene) into the abdominal cavity through the second abdominal segment. The needle was sterilized with acetone, ethanol, and locust saline solution between each injection. After 96 h of injection, the insect’s head and thorax were dissected. The dissected tissues were snap-frozen in liquid nitrogen and stored at −80°C. The RNA was extracted from the tissues, and the mRNA expression level of the targeted genes was quantified using RT-qPCR.

### Time Course of *dsRNase2* Knockdown

The effect of knocking down the mRNA expression of *dsRNase2* was studied across different time points (24, 48, 72, and 96 h) by injecting 10 µg of each ds*dsRNase2* and ds*GFP* into the treatment and the control group, respectively. The injections were performed as described above, and a total of 64 adult females (8 individuals per time point per gene) were used for the experiment. The midgut tissues were dissected 24, 48, 72, and 96 h after injections, and the mRNA expression level of *dsRNase2* was quantified using RT-qPCR. Similarly, the effect of an increased dose, 20 µg of *dsRNase2*, was also tested 72 h after injection using RT-qPCR.

### PLGA Nanoparticle Formulation and dsRNA Encapsulation

To prevent the degradation of dsRNA in the gut environment and to facilitate dsRNA uptake from the gut lumen, we encapsulated dsRNA inside PLGA nanoparticles using a double-emulsion (water in oil in water: w/o/w) method. The emulsion is a colloidal system of 2 or more liquids that are immiscible to each other (Vilela et al. 2018). Briefly, the dsRNA-dissolved aqueous solution was dissolved into an oil phase to make a water-in-oil formulation. Then, the w/o formulation was applied to a water phase to make a w/o/w formulation. dsRNA (100 µg) was emulsified in 1 ml dichloromethane (DCM) containing 100 mg PLGA using a probe ultrasonicator for 10 s on an ice bath. Primary emulsion was added to 2 ml 5% polyvinyl alcohol (PVA) solution, then sonicated for 10 s on an ice bath. The emulsion was transferred to the 0.25% PVA solution (100 ml) and stirred for 3 h to remove DCM. dsRNA-encapsulated PLGA nanoparticles were washed in MilliQ water 3 times and resuspended.

### Release Kinetics of PLGA Nanoparticle Within the Mormon Cricket After Oral Delivery

To characterize how the PLGA nanoparticles would release dsRNA inside the digestive system, we quantified the release kinetics of the nanoparticles. The PLGA nanoparticles hydrolyze in water, and the rate of hydrolysis inversely correlates with the molecular weight of the nanoparticles, such that higher molecular weight nanoparticles would release the encapsulated dsRNA slower. To study the release kinetics of nanoparticles of different molecular weights, we first encapsulated coumarin 6 dye as a proxy to dsRNA within the PLGA nanoparticles of 3 different molecular weights: 7–20 kDa (low) (11088-7-20k, Nanosoft Biotechnology LLC, NC), 50–70 kDa (medium) (11088-50-70k, Nanosoft Biotechnology LLC, NC), and 70–100 kDa (high) (11088 70-100k, Nanosoft Biotechnology LLC, NC). The particle size and size distribution were determined by dynamic light scattering using a particle size analyzer (ZetaPALS, Brookhaven Instruments, Holtsville, NY) (Supplementary Fig. 1). To prepare coumarin 6-loaded PLGA nanoparticles, coumarin 6 (1 mg) and PLGA (100 mg) in 1 ml DCM was added to 10 ml of 5% PVA solution. The mixture was sonicated for 10 s in ice and transferred to the 0.25% PVA solution (100 ml). The emulsion was stirred for 3 h. Coumarin 6-loaded nanoparticles were washed 3 times with MilliQ water and obtained by centrifugation. The 50 µl of the coumarin 6-encapsulating nanoparticles was applied onto lettuce leaf discs and allowed to dry for 15 min and then fed to 1-day starved adult females. The hemolymph was extracted from each individual at 6, 12, 24, 48, 72, 96, and 120 h after feeding (10 replicates per treatment). The traces of coumarin 6 dye in the hemolymph was quantified using a multimode plate reader (Molecular Devices Co, San Jose, CA) with an excitation wavelength of 440 nm and an emission wavelength of 534 nm.

### Comparison of the Effect of Oral Feeding of Naked dsRNA and dsRNA-Encapsulating Nanoparticles

To test whether encapsulating dsRNA inside the PLGA nanoparticles improved the RNAi efficiency, we compared the effect of oral feeding of 2 µg of naked dsRNA or dsRNA-encapsulating nanoparticle (high molecular weight) inducing the RNAi response. For each trial, single adult females were kept in individual plastic cups and starved for 1 day. The next day, 20 µl of dsRNA of respective targeted genes (*AnnIX*, *GAPDH*, *RpL5*) and control (*GFP*) were spread onto lettuce leaf discs, and the insects were allowed to feed on lettuce leaf discs. Once the insects entirely consumed the leaf discs, they were allowed to feed on MC food. The feeding experiment consisted of 80 individuals, with 10 individuals per gene for each time point. After 24 and 72 h of feeding, the head and thorax tissues were dissected and preserved in RNA*later* (Thermo Fisher Scientific, Waltham, MA). The samples were stored at −80°C until further processing for RNA extraction and RT-qPCR analysis.

### Dose–Dependent Response Assay After Oral Feeding of dsRNA-Encapsulating Nanoparticles

We tested various doses (5, 10, 15, 20, 25 µg) of nanoparticles (high molecular weight) encapsulating ds*CHS1* and ds*GAPDH* by oral feeding in inducing RNAi response. The feeding bioassay was performed as mentioned above. The tissues (head, thorax) were dissected and preserved for RNA extraction and RT-qPCR analysis 72 h after feeding. The increased dose of 60 µg of ds*GAPDH*-encapsulating nanoparticle was also tested. In this case, the dose was split into 2 subsequent doses: the first half dose, 30 µg was fed at 0 h, while the remaining half, 30 µg was fed at 24 h for treatment (ds*GAPDH*) and control (ds*GFP*) group respectively. The head and thorax tissues were dissected and preserved for RNA extraction and RT-qPCR analysis 96 h after the second feeding.

### dsRNA Stability Assay in the Fresh Midgut Juice

We tested the stability of dsRNA in the midgut juice to determine how long it would take for *dsRNase2* to degrade dsRNA. The abdomen was dissected, and midgut tissues were isolated from 5 adult females. The isolated midguts were then centrifuged at 16,000 g for 20 min at 4°C. The supernatant was collected and used for further analysis. The dsRNA stability was performed by incubating 2 µl of naked dsRNA or ds*GAPDH*-encapsulating nanoparticles (300 ng/µl) in 18 µl of fresh midgut juice at room temperature. The stability was assayed at different time points (1, 2, 5, and 10 min), and the samples were assessed on 1% agarose.

## Results

### Injection of ds*GAPDH* Showed Dose-Dependent Responses

**Fig. 1.**
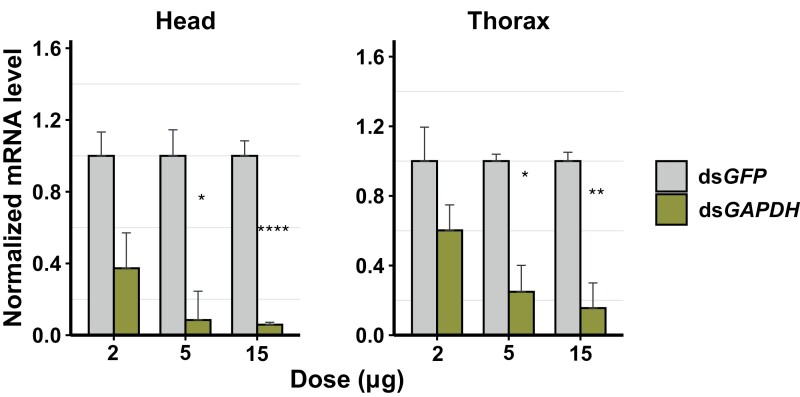
Dose-dependent response assay after the injection of ds*GAPDH* (2, 5, and 15 µg). The mRNA level of the targeted genes was measured in different tissues. Left panel: Head tissue; Right panel: Thorax tissue. Three to five biological replicates and 2 technical replicates were used for RT-qPCR. *EF1*α and *RpL5* were used as reference genes for normalization. The mRNA level in the treated (*GAPDH*) group was relative to the control (*GFP*) group at the same dose. Error bars indicate the standard error of mean. The statistical significance of differences was analyzed with Student’s *t*-test or Wilcoxon test. Significant differences between the control and target gene are indicated by asterisks (*: *P* < 0.05; **: *P* < 0.01; ****: *P* < 0.0001).

The injection of ds*GAPDH* resulted in mRNA knockdown across all of the doses we tested (Fig. 1). The increase in ds*GAPDH* dose resulted in a gradual increase in knockdown efficiency. For example, the injection of 2 µg ds*GAPDH* resulted in a 2–3-fold change in both tissue types, while the injection of 5 µg ds*GAPDH* resulted in an increase in knockdown in the head (12-fold change) and thorax (4-fold change) (Fig. 1). Similarly, the injection of 15 µg ds*GAPDH* resulted in even more significant knockdown in the head (16-fold change) and thorax (6-fold change) (Fig. 1).

### Injection of ds*dsRNase2* Did Not Cause Knockdown Across Different Time Points

**Fig. 2.**
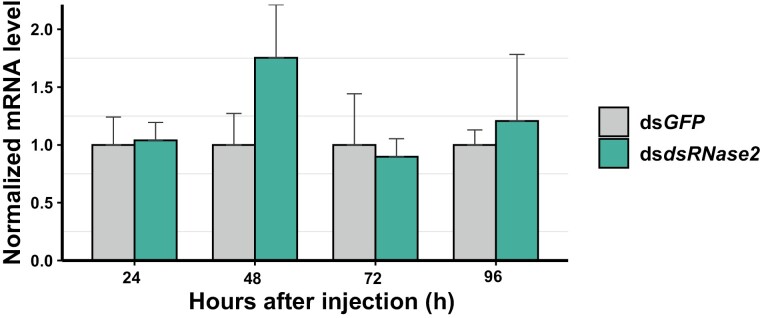
Time course assay after injection of 10 µg ds*dsRNase2*. The mRNA level of the targeted gene was measured in the midgut tissue for different time durations (24, 48, 72, and 96 h). Three to five biological replicates and 2 technical replicates were used for RT-qPCR. *EF1*α and *RpL5* were used as reference genes for normalization. The mRNA level in the treated (*dsRNase2*) group was relative to the control (*GFP*) group at the same time point. Error bars indicate the standard error of mean.

We tested whether we could knock down the mRNA expression level of *dsRNase2* in the midgut by injection, based on our positive results on systemic RNAi using ds*GAPDH* (Fig. 1). Because *dsRNase2* is mainly expressed in the midgut, we quantified the mRNA level in the midgut at 4 time points (24, 48, 72, and 96 h) after injection. Over the course of the experiment, we found that injecting ds*dsRNase2* did not cause knockdown, but the overexpression of *dsRNase2* was observed at 48 h instead (Fig. 2). The increased dose of 20 µg of ds*dsRNase2* resulted in a similar overexpression of *dsRNase2* at the studied time point, without causing a knockdown of *dsRNase2* (Supplementary Fig. 2).

### Release Kinetics of the PLGA Nanoparticles

**Fig. 3.**
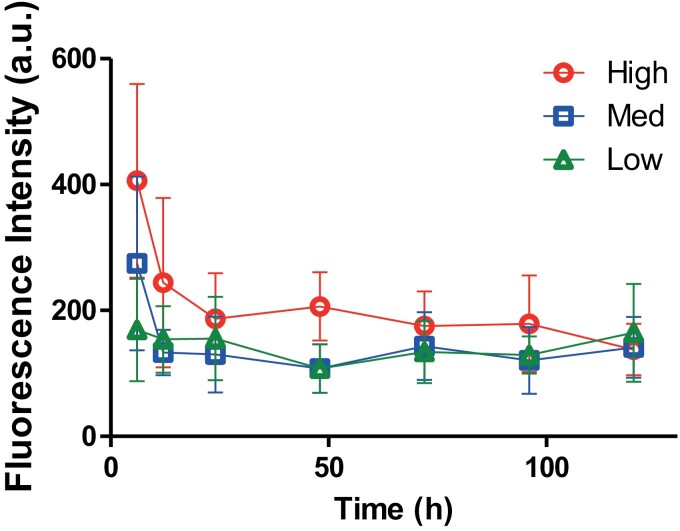
Release kinetics of PLGA nanoparticle encapsulated coumarin 6 in the hemolymph of Mormon cricket.

We found that the amount of coumarin 6 dye released in the first 6 h was the highest in the high molecular weight, followed by the medium and the low molecular weight (Fig. 3). Given that the release kinetics depended on the breaking of hydrogen bonds (hydrolysis), this pattern indicated that the low molecular weight nanoparticles started releasing the coumarin 6 dye nearly as soon as they were ingested. The medium molecular weight nanoparticles were slower in releasing the dye compared to the low molecular weight nanoparticles, but from 12 h and on, there was no difference between the 2, indicating that by 12 h the nanoparticles were completely hydrolyzed (Fig. 3). We found that the high molecular weight nanoparticles were slowest in releasing the dye. So, the nanoparticles were still releasing the coumarin 6 dye at 6 h, which continued to 12 h (Fig. 3). From 24 h, most of the nanoparticles appeared to be hydrolyzed, with not much difference among the 3 types of nanoparticles (Fig. 3). Based on this result, we found that the low molecular weight nanoparticles released most of the encapsulated coumarin 6 dye within the first 6 h after oral delivery, the medium released most of the encapsulated content within the first 12 h, and the high continued to release the content for at least 24 h after oral delivery (Fig. 3).

### Effect of Oral Feeding of Naked dsRNA or dsRNA-Encapsulating Nanoparticles

**Fig. 4.**
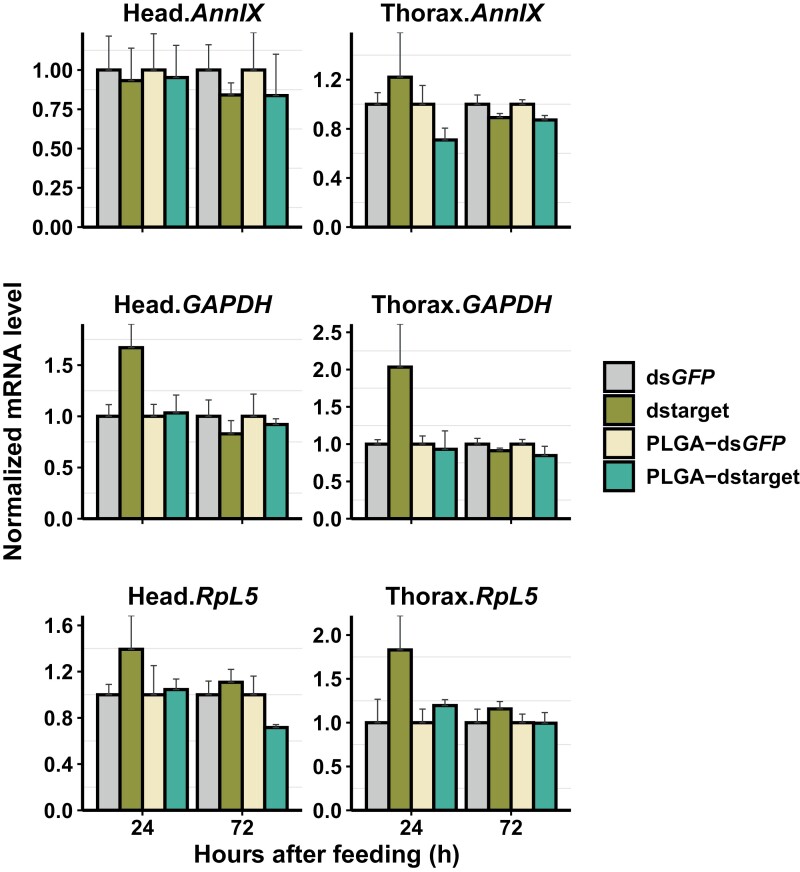
Effect of oral feeding of naked dsRNA or dsRNA-encapsulating nanoparticles (2 µg) of 3 targeted genes (*AnnIX*, *GAPDH*, *RpL5*) in different tissues at 24 and 72 h. Left panel: Head tissue; Right panel: Thorax tissue. Row panels represent targeted genes, and the bars represent different treatment groups, ds*GFP*, dstarget (*AnnIX*, *GAPDH*, *RpL5*), PLGA-ds*GFP*, and PLGA-dstarget. Three to five biological replicates and 2 technical replicates were used for RT-qPCR. *EF1*α and *Act5C* were used as reference genes for normalization. The mRNA level in the treated group was relative to the control (*GFP*) group at the same dose. Error bars indicate the standard error of mean.

For all the targeted genes, the oral feeding of naked dsRNA or dsRNA-encapsulating nanoparticles did not significantly affect the mRNA expression level (Fig. 4). However, some level of knockdown was observed at some time points (mostly 72 h) after feeding the dsRNA-encapsulating nanoparticles (Fig. 4). For example, in the head tissue, *RpL5* showed a nearly 1.5-fold change at 72 h after feeding, while in the thorax tissue, *AnnIX* showed a nearly 1.5-fold change at 24 h after feeding (Fig. 4). Interestingly, some gene-specific and tissue-specific overexpression patterns were also observed after feeding the naked dsRNA (Fig. 4). *GAPDH* and *RpL5* overexpressed at 24 h, which was consistent in both tissue types after feeding naked ds*GAPDH* and ds*RpL5,* respectively, compared to the control (*GFP*) group (Fig. 4). *AnnIX* also showed some level of overexpression at 24 h in thorax tissue after feeding the naked ds*AnnIX* (Fig. 4).

### Rapid Degradation of dsRNA in the Midgut Juice

**Fig. 5.**
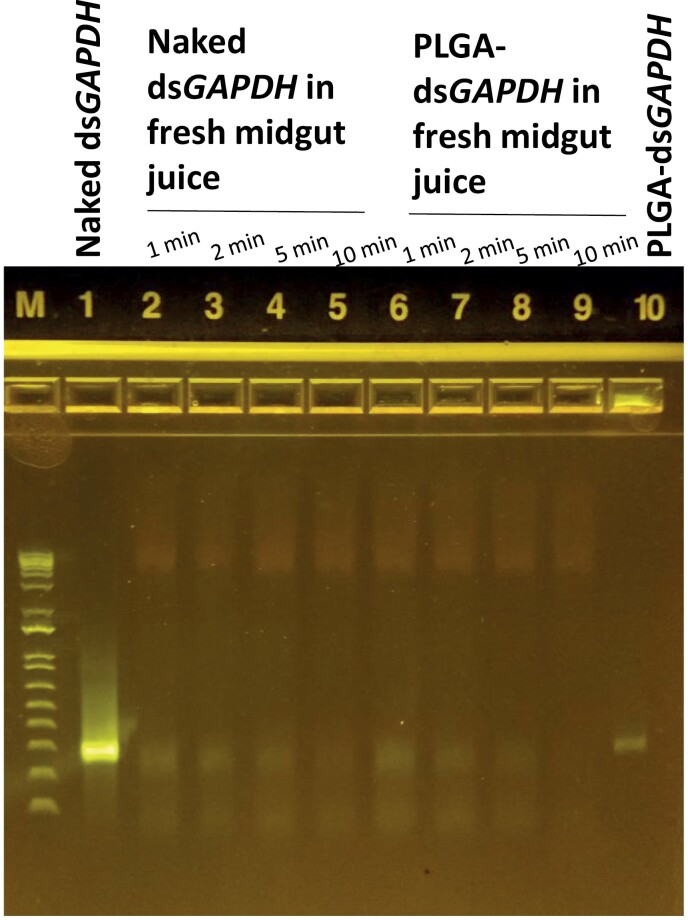
Naked ds*GAPDH* and ds*GAPDH*-encapsulating nanoparticle stability assay in the fresh midgut juice.

The *ex vivo* dsRNA stability assay showed the degradation of both naked dsRNA and dsRNA-encapsulating nanoparticles within 10 min when incubated in the fresh midgut juice (Fig. 5), indicating the presence of dsRNA-degrading enzymes and the instability of the nanoparticles in the midgut juice.

### Dose-Dependent Response Assay After Oral Feeding of dsRNA-Encapsulating Nanoparticles

**Fig. 6.**
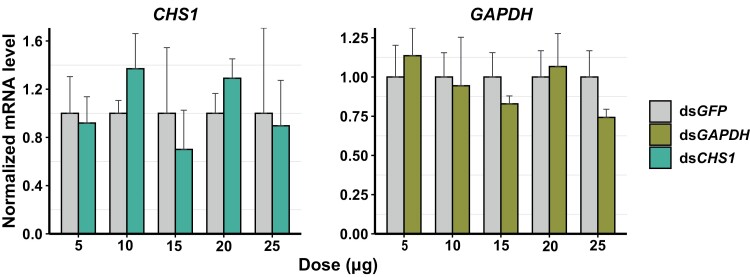
Dose-dependent response assay after oral feeding of dsRNA-encapsulating nanoparticles, ds*CHS1*, and ds*GAPDH* (5, 10, 15, 20, and 25 µg). The mRNA level of the targeted genes was measured in the head tissue. Left panel: *CHS1* gene; Right panel: *GAPDH* gene. Three to five biological replicates and 2 technical replicates were used for RT-qPCR. *EF1*α and *RpL5* were used as reference genes for normalization. The mRNA level in the treated *(CHS1*/ *GAPDH*) group was relative to the control (*GFP*) group at the same dose. Error bars indicate the standard error of mean.

To rule out the dose constraint with the previous feeding experiment, we tested different doses of dsRNA-encapsulating nanoparticles, targeting 2 different genes, *CHS1* and *GAPDH* (Fig. 6). Although not significantly different, we found some level of knockdown in both targeted genes after feeding 15 and 25 µg of dsRNA-encapsulating nanoparticles (Fig. 6). While other studied doses did not show a knockdown effect, both genes showed an overexpression at some doses instead (Fig. 6). The increased dose of 60 µg of ds*GAPDH*-encapsulating nanoparticles also did not elicit knockdown effects while showing a similar overexpression (Supplementary Fig. 3).

## Discussion

The non-toxic and species-specific nature of RNAi provides an attractive alternative to chemical insecticides for insect pest management. However, the key limitation of this technology is that not all insect orders respond equally well to RNAi. Generally, coleopterans are reported to be highly sensitive to RNAi (Baum et al. 2007), while orthopterans, hemipterans, dipterans, and lepidopterans showed high variability between and within species (Terenius et al. 2011, Baum and Roberts 2014, Wang et al. 2016, Rana et al. 2020). RNAi delivery methods also affect the RNAi efficiency, with variable responses in several insect species (Cooper et al. 2019). In general, the injection yields an effective knockdown in most insect orders, but the dose of dsRNA required to elicit RNAi responses varies from a few ng to 100 µg. Coleopteran species only require low doses to induce a knockdown compared to other insect orders (Baum et al. 2007), while lepidopterans require high doses to induce a knockdown (Terenius et al. 2011). In Orthoptera, the desert locust is shown to exhibit a high systemic RNAi response with a very low dose of dsRNA (1 ng), leading to a significant knockdown (Wynant et al. 2012). Although in the same order as the desert locust, the Mormon cricket shows a distinctly different pattern, requiring a very high dose of dsRNA (5 µg) to induce a significant knockdown. The reason why some insect species require a high dose and others need less is still unclear, but it may have to do with the efficiency of the dsRNA uptake mechanism, the expression of RNAi core machinery, and/or the activity of nuclease-degrading enzymes, as well as the body size. Unlike plants and nematodes, the orthologs of RNA-dependent RNA polymerase (RdRp) have not yet been found in insects to assess their contribution to these variable responses.

Responses to the oral delivery of dsRNA can also vary at the order level. Coleopterans are known to exhibit variable responses to oral delivery of dsRNA. For example, *D. virgifera* is sensitive to both injection and feeding, while *Anthonomus grandis* (boll weevil) is sensitive to injections but not feeding (Gillet et al. 2017). Similarly, hemipteran species, such as *Halyomorpha halys* (brown marmorated stink bug) is sensitive to both injection and feeding (Mogilicherla et al. 2018), while *Lygus lineolaris* (tarnished plant bug) is sensitive to injection but not feeding (Allen and Walker 2012). Likewise, most of the studied lepidopteran species also showed variable responses to different delivery methods (Terenius et al. 2011). For Orthoptera, the oral delivery of dsRNA is not known to induce any RNAi responses.

In this study, we have demonstrated that the RNAi responses in the Mormon cricket differ between the delivery methods. The injection of dsRNA results in an effective knockdown, but the responses vary among tissues, targeted genes, and the dosage of dsRNA. For example, the injection of ds*GAPDH* resulted in a high knockdown in the head compared to the thorax. On the contrary, the injection of ds*dsRNase2* did not show any knockdown in the midgut across different time points. One possible explanation for this pattern might be an inefficient dsRNA uptake by the midgut epithelial cells, causing refractory responses in the midgut tissue. These results are congruent with the earlier reports on locusts where tissue-specific responses were found (Wynant et al. 2012, Ren et al. 2014, Spit et al. 2017). The oral delivery of dsRNA did not result in a knockdown regardless of the tissues, genes, and doses, strongly indicating that the RNAi response is refractory to the oral delivery.

The stability of dsRNA after oral delivery can be affected by several factors, one of which is the degradation of dsRNA by the *dsRNases*, which has been reported among various insect orders (Singh et al. 2017). In locusts, the *dsRNase2* is expressed predominantly in the midgut (Wynant et al. 2014, Song et al. 2019), and its role in degrading dsRNA in insects is well known (Arimatsu et al. 2007). Although knocking down *dsRNase2* in some insect pests resulted in high RNAi efficiency (Song et al. 2017, Spit et al. 2017), our initial attempt to eliminate the *dsRNase* activity by knocking down *dsRNase2* did not work well. This might be due to the reason suggested above or there might be other paralogous *dsRNase* genes present in the midgut that are not identified in the study. Based on these observations, we speculate that the paralogous genes, based on their sequence similarity and substrate specificity, may compensate for the loss of function of *dsRNase2*. Hence, individual knockdown of all paralogous genes is essential to derive a clear conclusion. In addition, the knockdown of any other midgut specific gene would be important for answering whether this response is specific to *dsRNase2*.

The degradation of dsRNA might also be due to other factors of the gut environment, including the pH. For example, the extremely alkaline environment in the lepidopteran gut system (Dow 1984) is often related to dsRNA instability and the resulting low RNAi efficiency. However, while orthopterans do not have an extremely alkaline midgut they do have fluctuating pH ranges from 5.0 to 7.5 in different regions of the gut, depending on the presence or absence of food (Uvarov 1966). In the Mormon cricket, similar variable pH conditions might affect the stability of dsRNA or the activity of the nuclease enzymes, as reported in the migratory locust (Luo et al. 2013).

Alternatively, the dsRNA uptake mechanism is also not explored yet in the Mormon cricket and there is no clear evidence showing the passing of dsRNA from the gut lumen. However, in desert locusts, an endocytosis-mediated uptake mechanism with injection is reported (Ren et al. 2014, Wynant et al. 2014b). The mechanism may not necessarily be the same with the feeding or which might not even be present in the Mormon cricket digestive system. More efforts to identify and study the dsRNA uptake from the gut in the Mormon cricket and other orthopteran species are crucial to acquire further insights.

To overcome the dsRNA degradation in the digestive system, we tested a novel nanoparticle that had not yet been applied to encapsulate dsRNA in insect systems. The PLGA is a well-characterized polymer that has been extensively used for drug and protein delivery to humans (Wise et al. 1976, Gilding and Reed 1979, Makadia and Siegel 2011, ECRI 2020), and because it has already been approved by the FDA, we initially thought that it would be a good fit as a carrier for dsRNA for insect pests. We started with characterizing the release kinetics of the PLGA nanoparticles and, as expected, the high molecular weight nanoparticles hydrolyzed the slowest, releasing the encapsulated dye slowly. This was a promising result because the observed pattern suggested that the high molecular weight PLGA nanoparticles could protect dsRNA long enough before the potent degradation by the dsRNases would start. We found that the coumarin 6 dye accumulated in the hemolymph in a consistent manner, which could either mean that the nanoparticles were able to pass through the gut membrane to reach the hemolymph and hydrolyze to release the dye, or that the nanoparticles hydrolyzed inside the gut, and the released coumarin 6 dye passed through the gut membrane to reach the hemolymph.

Based on the release kinetics that we quantified, we formulated dsRNA-encapsulated nanoparticles with the high molecular weight PLGA and fed them to the Mormon cricket to see if an RNAi response could be observed. However, the dsRNA-encapsulating nanoparticles failed to induce a strong RNAi response after oral feeding. Furthermore, even when higher doses of dsRNA-encapsulating nanoparticles were orally fed, we did not observe any significant knockdown effects. One explanation for this observation could be that the nanoparticles hydrolyzed inside the digestive system, releasing the dsRNAs, which would be quickly degraded by the dsRNases present in the midgut. Our *ex vivo* dsRNA stability assay showed rapid degradation of dsRNA encapsulated in the nanoparticles in the fresh midgut juice, which suggests that the highly aqueous environment within the midgut compromises the stability of the nanoparticles. Another possibility could be that the dsRNA uptake mechanism could be different in the gut than in other tissues. The Mormon cricket does show systematic RNAi via injection, which means that dsRNA can be easily taken up in other tissues from outside, but perhaps there is a barrier in the gut that prevents dsRNA uptake.

Generally, knockdown of targeted genes is the expected outcome in typical RNAi experiments aimed to devise a pest management strategy unless the targeted genes are involved in some kind of immune response or the core RNAi machinery (Lee et al. 2006, Guan et al. 2018). However, some genes that are not known to be directly involved in such functions may sometimes show overexpression in response to the injected or fed dsRNAs. Interestingly, in our experiments, we also found some level of overexpression (though statistically insignificant) of the targeted genes in some experiments. Mostly, the overexpression was observed in the early time points compared to the later time points. For example, the *GAPDH* and *RpL5* overexpressed after 24 h of feeding naked dsRNA compared to the control (*GFP*) group, indicating some kind of immune response that is specifically triggered by the dsRNAs of the respective endogenous target genes rather than dsRNA of the exogenous *GFP* gene. In organisms such as nematodes, 2 nonoverlapping RNAi pathways exist: endogenous and exogenous pathways. The exogenous pathway utilizes the RNAi machinery components, thereby limiting their availability for the endogenous pathway, which may result in overexpression of endogenous genes (Lee et al. 2006). This might explain the overexpression of some genes observed in our experiments and may also explain the absence of overexpression for the control *GFP* gene. Besides, *GAPDH* and *RpL5* may play a role in stress response where their direct involvement is still unknown. On the other hand, the increase in *dsRNase2* expression at 48 h could also be the result of similar cross regulation between the 2 pathways. Of course, the increase of *dsRNase2* also confirms that *dsRNase2* is likely involved in the immune response against dsRNA/viruses (Nestle and Roberts 1969). In addition, we also suspect the presence of paralogs for our targeted *dsRNase* as explained previously.

In conclusion, this is the first study demonstrating the different responses of injection and oral feeding of dsRNA in the Mormon cricket. Injections of naked dsRNA showed dose-dependent, tissue-specific, and gene-specific responses, while the feeding of naked or dsRNA-encapsulating nanoparticles showed refractory response regardless of the dose, tissue, or gene, which we think is order-specific. Studying more species in Orthoptera is essential to understand the underlying mechanism behind the refractory nature of the species’ response to oral feeding of dsRNA. The different response could be due to the presence of dsRNA-degrading enzymes in the midgut, which are reported in the study. The degradation of dsRNA-encapsulating nanoparticles and their uptake mechanism could most likely be optimized by developing more efficient nanoparticles, which would be a way forward towards devising alternative management strategies for the Mormon cricket and other orthopteran pests.

## Supplementary Material

iead063_suppl_Supplementary_Figure_S1

iead063_suppl_Supplementary_Figure_S2

iead063_suppl_Supplementary_Figure_S3
