# Supplementary figures and images for: Differential responses to double-stranded RNA injection and feeding in Mormon cricket (Orthoptera: Tettigoniidae)
Source: J Insect Sci. 2023 Aug 1;23(4):10. doi: 10.1093/jisesa/iead063 (PMC10393271; doi:10.1093/jisesa/iead063)

PLGA mw: 7-20 k

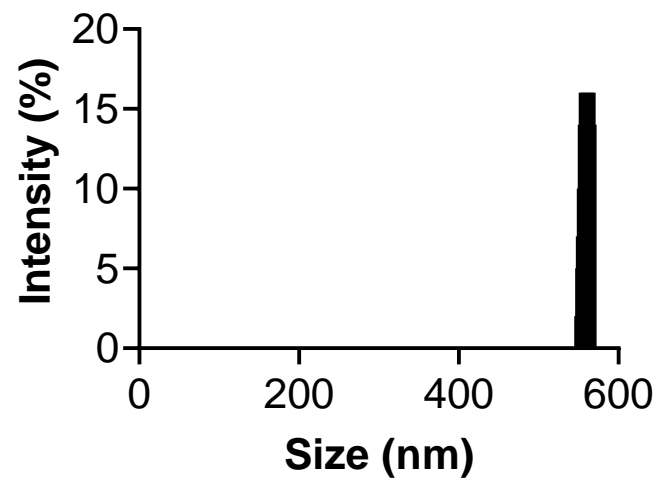

Mean diameter      559.7 nm

PLGA mw: 50-70 k

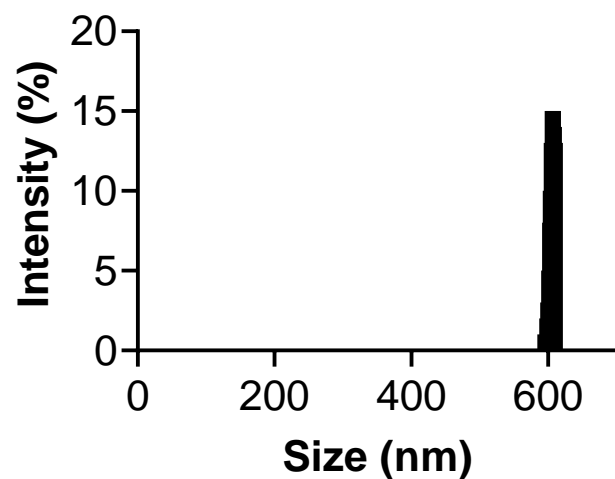

605.6 nm

PLGA mw: 70-100 k

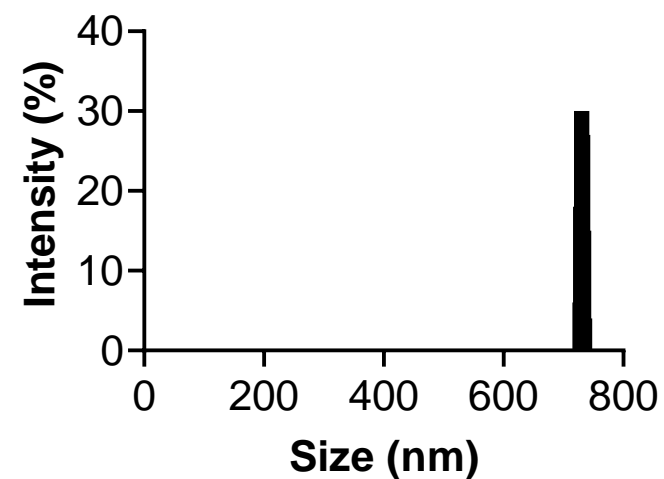

731.2 nm

Supplement: iead063_suppl_Supplementary_Figure_S1 [file iead063_suppl_supplementary_figure_s1.pdf]

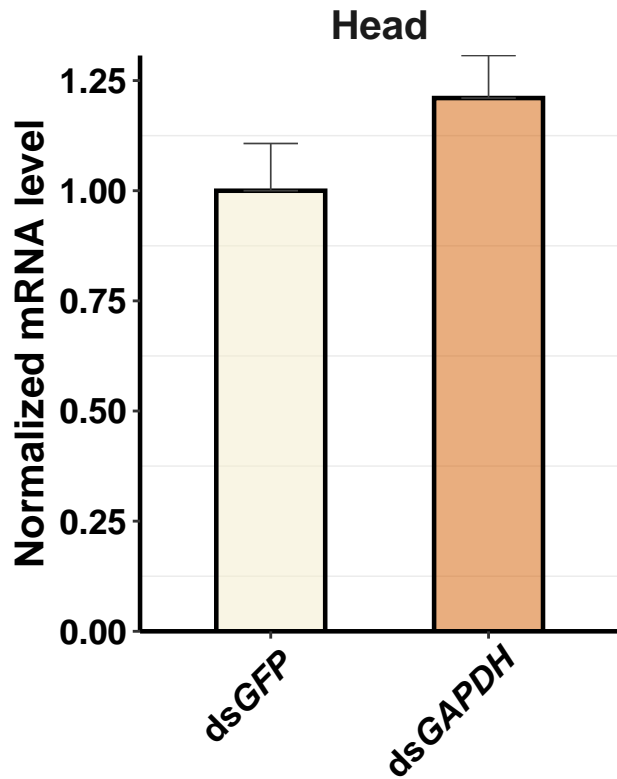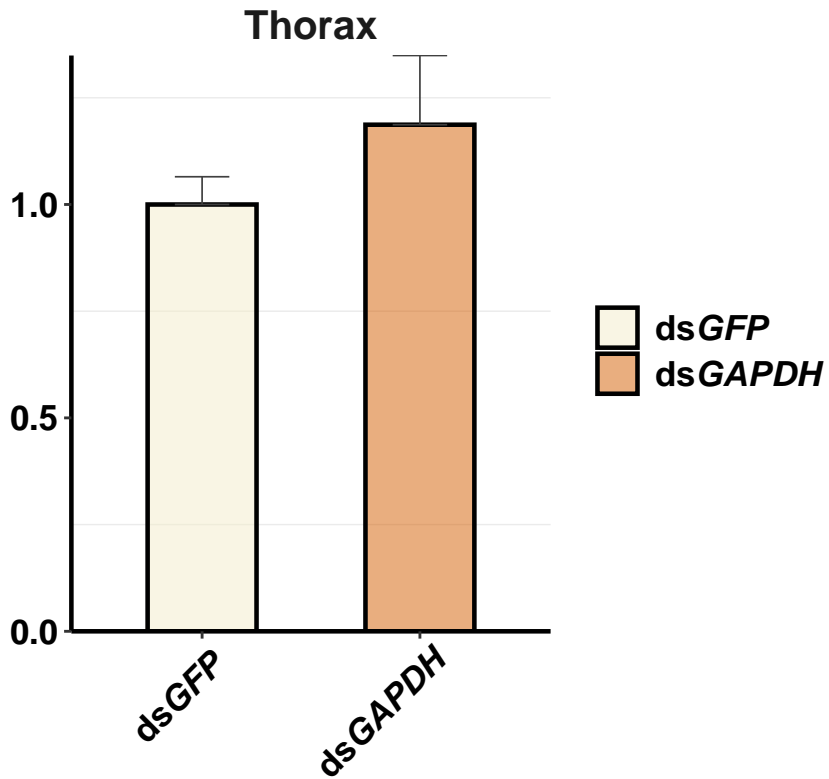

Supplement: iead063_suppl_Supplementary_Figure_S2 [file iead063_suppl_supplementary_figure_s2.pdf]

# *dsRNase2*

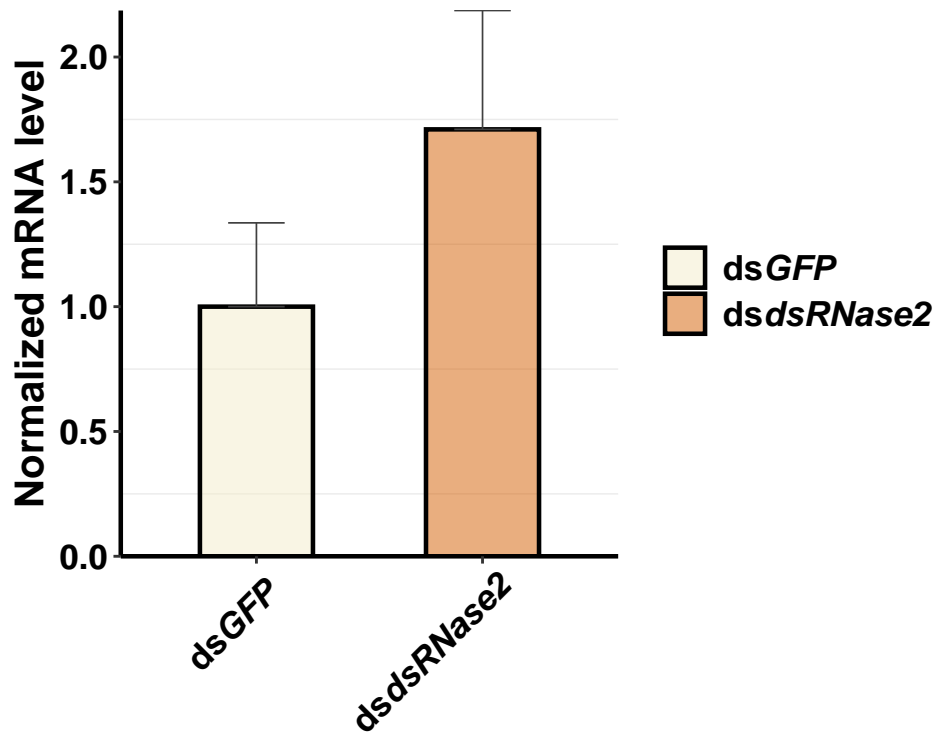

Supplement: iead063_suppl_Supplementary_Figure_S3 [file iead063_suppl_supplementary_figure_s3.pdf]
